# Development and Validation of a Nomogram for Differentiating Combined Hepatocellular Cholangiocarcinoma From Intrahepatic Cholangiocarcinoma

**DOI:** 10.3389/fonc.2020.598433

**Published:** 2020-12-09

**Authors:** Tao Wang, Wanxiang Wang, Jinfu Zhang, Xianwei Yang, Shu Shen, Wentao Wang

**Affiliations:** ^1^ Department of Liver Surgery and Liver Transplantation Center, West China Hospital of Sichuan University, Chengdu, China; ^2^ Department of Hepatobiliary, Pancreatic, and Splenic Surgery, The Affiliated Hospital of Inner Mongolia Medical University, Hohhot, China

**Keywords:** intrahepatic cholangiocarcinoma, combined hepatocellular cholangiocarcinoma, differential diagnosis, least absolute shrinkage and selection operator regression, nomogram

## Abstract

**Objectives:**

To establish a nomogram based on preoperative laboratory study variables using least absolute shrinkage and selection operator (LASSO) regression for differentiating combined hepatocellular cholangiocarcinoma (cHCC) from intrahepatic cholangiocarcinoma (iCCA).

**Methods:**

We performed a retrospective analysis of iCCA and cHCC patients who underwent liver resection. Blood signatures were established using LASSO regression, and then, the clinical risk factors based on the multivariate logistic regression and blood signatures were combined to establish a nomogram for a differential preoperative diagnosis between iCCA and cHCC. The differential accuracy ability of the nomogram was determined by Harrell’s index (C-index) and decision curve analysis, and the results were validated using a validation set. Furthermore, patients were categorized into two groups according to the optimal cut-off values of the nomogram-based scores, and their survival differences were assessed using Kaplan-Meier curves.

**Results:**

A total of 587 patients who underwent curative liver resection for iCCA or cHCC between January 2008 and December 2017 at West China Hospital were enrolled in this study. The cHCC score was based on the personalized levels of the seven laboratory study variables. On multivariate logistic analysis, the independent factors for distinguishing cHCC were age, sex, biliary duct stones, and portal hypertension, all of which were incorporated into the nomogram combined with the cHCC-score. The nomogram had a good discriminating capability, with a C-index of 0.796 (95% CI, 0.752–0.840). The calibration plot for distinguishing cHCC from iCCA showed optimal agreement between the nomogram prediction and actual observation in the training and validation sets. The decision curves indicated significant clinical usefulness.

**Conclusion:**

The nomogram showed good accuracy for the differential diagnosis between iCCA and cHCC preoperatively, and therapeutic decisions would improve if it was applied in clinical practice.

## Highlights

cHCC is a rare, distinct entity different from iCCA. Using the clinical data obtained from West China Hospital, the authors discovered that the prognosis of the cHCC was significantly worse than that of iCCA. The novel validated nomogram presented herein is a tool that can effectively differentiate cHCC from iCCA preoperatively.

## Introduction

Intrahepatic cholangiocarcinoma (iCCA) is the second most common primary liver cancer after hepatocellular carcinoma (1, 2). Combined hepatocellular cholangiocarcinoma (cHCC) is a rare malignant liver tumor containing components of both hepatocellular carcinoma (HCC) and intrahepatic cholangiocarcinoma (iCCA) (3, 4),accounting for 0.8%–14.3% of primary liver malignancies, with incidences widely varying among studies (5–7). Previous studies have classified cHCC and iCCA in the same category (8–10), but there is controversy about their clinical features and prognoses; for example, some studies have suggested that patients with cHCC have a poorer prognosis than those with iCCA (5, 11, 12), while other studies have reported the opposite conclusion (13).

An accurate differential diagnosis of cHCC and iCCA before surgery remains an important goal with prognostic significance because of differences in therapeutic strategies and prognoses between two; however, at present, the gold standard for cHCC diagnosis is still fine needle aspiration biopsy or a histopathological examination after surgery. With the development of radiological technology, there may be some features of imaging that imply cHCC; however, when cHCC has characteristics consistent with cholangiocarcinoma differentiation in variable proportion, cHCC is often easily misdiagnosed as iCCA (7, 14, 15). Thus, better preoperative noninvasive prediction models are needed to differentiate cHCC from iCCA. We retrospectively performed a comprehensive analysis of the clinicopathological characteristics and survival information of cHCC and iCCA patients in our single center. Furthermore, we established a feasible and straightforward simplified nomogram based on laboratory study variables selected by the least absolute shrinkage and selection operator (LASSO) regression analysis as well as other clinical risks obtained by multivariate logistic regression for the preoperative differential diagnosis between cHCC and iCCA. LASSO regression analysis was used to reduce high-dimensional data and choose the predictive factors in the differential diagnosis of cHCC and iCCA (16, 17).

## Methods

### Patients and Study Design

**Figure 1:**
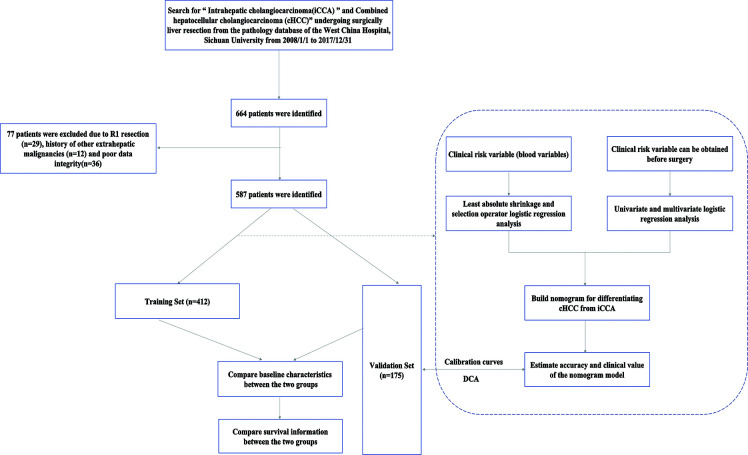
The flowchart of patient selection.

This retrospective study was conducted on iCCA and cHCC patients who underwent curative liver resection between January 2008 and December 2017. Our selection criteria for patients in this study included the following (1) age ≥ 18 years (2); patients who underwent R0 resection, defined as the absence of microscopic or gross residual disease, pathology of the resection margin is was confirmed to be negative, and after the organ or tissue directly invaded by the tumor was combined with resection, the surgical margin was also negative (3); contrast-enhanced CT of the abdomen and laboratory study were performed less than 1 week prior to surgery; and (4) detailed clinical characteristics. Our exclusion criteria for this study were as follows:(1) postoperative pathology confirmed hepatocellular carcinoma (HCC) and R1 excision or tumor margin was not specified in detail (2); the patient had a history of other extrahepatic malignancies; and (3) poor clinical data integrity. In this study, the whole set was randomly divided into two sets: the training set(n=412, 70%) and the validation set (n=175, 30%). The flowchart of the present study selection is shown in **Figure 1** and the clinicopathologic characteristics of patients in the training and validation sets are listed in **Table 1**. This study was conducted in accordance with the Declaration of Helsinki and was approved by the ethics committee of Sichuan University West China Hospital. Informed consent was obtained from all the patients.

**Table 1:** Baseline characteristics of patients in intrahepatic cholangiocarcinoma (iCCA) and combined hepatocellular cholangiocarcinoma (cHCC).

Variables	Patients					
	Training set			Validation set		
	iCCA group(n=280)	cHCC group(n=132)	*P-value*	iCCA group(n=118)	cHCC group(n=57)	*P-value*
**Age (years), median (IQR)**	57(48–64)	52(45–59)	0.005	58(50–64)	55(46.25–58)	0.004
**Gender (male/female),**	151/129	105/27	<0.001	55/63	50/7	<0.001
**Portal hypertension, n (%)**			0.003			<0.001
Yes	40(14.29%)	35(26.52%)		13(11.02%)	21(36.84%)	
No	240(85.71%)	97(73.48%)		105(88.98%)	36(63.16%)	
**HBsAg positive, n (%)**			<0.001			<0.001
Yes	88(31.43%)	105(79.55%)		38(32.20%)	40(70.18%)	
No	192(68.57%)	27(20.45%)		80(67.80%)	17(29.82%)	
**Diabetes, n (%)**			0.700			0.906
Yes	40(14.3%)	17(12.9%)		11(9.3%)	5(8.8%)	
No	240(85.7%)	115(87.1%)		107(90.7%)	52(91.2%)	
**Biliary duct stones, n (%)**			<0.001			0.024
Yes	45(16.1%)	5(3.8%)		21(17.8%)	3(5.3%)	
No	235(83.9%)	127(96.2%)		97(82.2%)	54(94.7%)	
**Baseline laboratory investigations**						
WBC count ×10^9^/L, median (IQR)	6.62(5.42–8.00)	5.78(4.74–7.16)	0.005	6.31(5.15–7.76)	5.76(4.56–7.80)	0.177
NEUT count ×10^9^/L, median (IQR)	4.24(3.33–5.65)	3.62(2.87–5.11)	0.018	3.94(3.06–5.37)	3.55(2.85–5.13)	0.068
PLT count ×109/L, median (IQR)	163.5(122–219)	142.5(99.25–192.5)	0.015	132(132–218.75)	139(83–15.5)	0.011
ALT (U/L), median (IQR)	30(18–47)	33(25–49)	0.033	24.5(16–36)	38(26.5–58)	<0.001
AST (U/L), median (IQR)	30(23–42)	35.5(27–53)	0.007	28.5(23–37)	35(27.5–50.5)	<0.001
GGT (U/L), median (IQR)	66(36–132.25)	71(41–135)	0.598	66(22.75–136.5)	61(38–120)	0.360
TBIL (umol/L), median (IQR)	13.45(9.93–17.18)	13.35(10.23–18.08)	0.970	11.7(9.38–14.73)	13.3(9.50–20.4)	0.148
ALB (g/L), median (IQR)	42.5(39.7–45.1)	41.7(38.03–44.5)	0.274	42.7(40.28–45.93)	42.8(39.35–46.6)	0.958
PT(s), median (IQR)	11.6(11.0–12.3)	12.0(11.4–12.9)	0.017	11.5(11–12.13)	11.9(11.3–13)	0.037
INR, median (IQR)	1.02(0.96–1.08)	1.05(1.01–1.13)	0.001	1.0(0.95–1.06)	1.04(0.98–1.15)	0.001
AFP, ng/ml median (IQR)	3.18(2.26–5.37)	75.84(6.35–621)	<0.001	3.24(2.16–5.68)	44.09(3.71–226.10)	<0.001
CA19-9 level(U/mL), median (IQR)	78.19(19.28–861.35)	31.17(14.32–95.89)	<0.001	57.13(13.77–493.98)	23.49(13.64–47.61)	<0.001
**Tumor size (cm), median (range)**	5.5(4.2–8)	5.5(3.7–7.8)	0.448	6(4.6–8.0)	5.3(3.1–7.05)	0.098
**Tumor number (Multiple/solitary),**			0.317			0.001
multiple	90(32.14%)	36(27.27%)		39(33.1%)	6(10.5%)	
solitary	190(67.86%)	96(72.73%)		79(66.9%)	51(89.5%)	
**Tumor location**			<0.001			0.004
Left lobe	116(41.43%)	28(21.21%)		46(39.0%)	21(36.8%)	
Right lobe	103(36.79%)	84(63.64%)		44(37.3%)	33(57.9%)	
Both lobes	61(21.78%)	20(15.15%)		28(23.7%)	3(5.3%)	
**Extent of liver resection, n(%)**			0.082			0.342
major	166(59.29%)	90(68.18%)		81(68.64%)	35(61.40%)	
minor	114(40.71%)	42(31.82%)		37(31.36%)	22(38.60%)	
**MVI, n (%)**			0.002			0.001
Yes	52(18.57%)	43(32.58%)		21(17.80%)	23(40.35%)	
No	228(81.43%)	89(67.42%)		97(82.20%)	34(59.65%)	
**Macroscopic vascular invasion, n(%)**			0.205			0.622
Yes	96(34.29%)	37(28.03%)		35(29.66%)	19(33.33%)	
No	184(65.71%)	95(71.97%)		83(70.34%)	38(66.67%)	
**Satellite nodules, n(%)**			0.411			0.432
Yes	42(15%)	24(18.18%)		21(17.80%)	13(22.81%)	
No	238(850%)	108(81.82%)		97(83.20%)	44(77.19%)	
**Lymph node metastasis, n (%)**			0.024			0.027
Present	70(25%)	20(15.15%)		32(27.12%)	7(12.28%)	
Absent	210(75%)	112(84.85%)		86(72.88%)	50(87.72%)	
**Tumor encapsulation, n (%),**			0.759			0.088
incomplete	153(54.64%)	70(53.03%)		74(62.71%)	28(49.12%)	
complete	127(45.36%)	62(46.97%)		44(37.29%)	29(50.88%)	

HBsAg, hepatitis B surface antigen; WBC, white blood cell; NEU, neutrophil; PLT, platelet; ALT, alanine aminotransferase; AST, aspartate transaminase; GGT, γ-glutamyl transferase; TBIL, total bilirubin; ALB, albumin; PT, Prothrombin time; INR, international normalized ratio; AFP, alpha fetoprotein; CA19-9, carbohydrate antigen 19-9; MVI, microvascular invasion.

### Data Collection and Follow-Up

The clinical medical data of cHCC and iCCA patients who underwent curative liver resection were retrospectively collected from our hospital and included demographics, comorbid illnesses, portal hypertension, preoperative routine blood tests, biochemistry tests, tumor marker tests, tumor imaging data and survival information. In general, all patients who received curative liver resection were prospectively followed up through outpatient clinic visits or telephone calls at intervals of 2–3 months during the first year after the operation and 3–6 months thereafter. Chest CT examination, bone scintigraphy and PET-CT were performed when extrahepatic tumor recurrence was suspected. Oncological survival outcomes, including overall survival (OS) and recurrence-free survival (RFS), were collected until December 31, 2019. OS was defined as the interval between resection and death, or the period up to the last follow-up. RFS was defined as the interval from after surgery to tumor recurrence, including intrahepatic tumor recurrence and extrahepatic metastasis, or the period up to the last observation endpoint.

### Risk Factors for Presence of cHCC

Univariate logistic regression analysis was performed to estimate the impacts of demographics, comorbid illnesses and imaging features on distinguishing cHCC and iCCA in the training set. Stepwise multivariate logistic regression analysis was further performed to screen for independent risk factors at a significant level. The LASSO logistic regression model was used to build a prognostic classifier, which integrated all types of laboratory study variables that can be obtained before surgery, to differentiate iCCA from cHCC in the training set. Using the coefficients derived from the LASSO logistic regression models, we then constructed a formula to calculate a score for each patient. We used the receiver operating characteristic (ROC) curve with calculations of the area under the curve (AUC) to determine the optimal cut-off value of the blood signature score. Restricted cubic spline (RCS) was used to evaluate the relationship between the blood signature score obtained by LASSO logistic regression and the outcome of distinguishing cHCC from iCCA.

### Construction, Assessment, and Internal Validation of Nomograms

Laboratory study variables chosen by LASSO regression and the results of multivariate logistic regression were included in the model. All possible diagnostic factors are performed to construct a simplified nomogram for the differential diagnosis of iCCA and cHCC. The differential accuracy of the models was measured using the C-index, quantifying the level of agreement between the predicted probabilities and the actual possibility of having the event of interest, and the bootstrap estimate of slope shrinkage (18). The bootstrap resampling method was chosen for the internal validation of the predictive models’ selecting 1000 repetitions. Decision curve analysis (DCA) was used to determine the clinical application value of the nomogram by evaluating the net benefit (19). Clinical impact curves were further drawn to evaluate the clinical impact of the nomogram to help understand its importance more intuitively (20).

### Statistical Analysis

The Mann-Whitney U test was used to compare continuous variables between two patient groups. The chi-squared test and two-tailed Fisher’s exact test were used for the comparison of categorical variables between two groups. Continuous variables are expressed as the medians and interquartile ranges(*Q1-Q3*), and categorical variables are expressed as the numbers and percentages. R version 4.0.0 (http://www.r-project.org/) was used for ROC curve analysis, RCS, LASSO logistic regression, nomogram generation, C-index assessment, calibration plot generation, and DCA. The rest of the analyses were conducted using SPSS statistical software version 25.0 (IBM Corporation, Armonk, NY). In all analyses, *P* < 0.05 was considered to indicate statistical significance.

## Results

### Clinical Characteristics of the Study Patients

**Figure 2:**
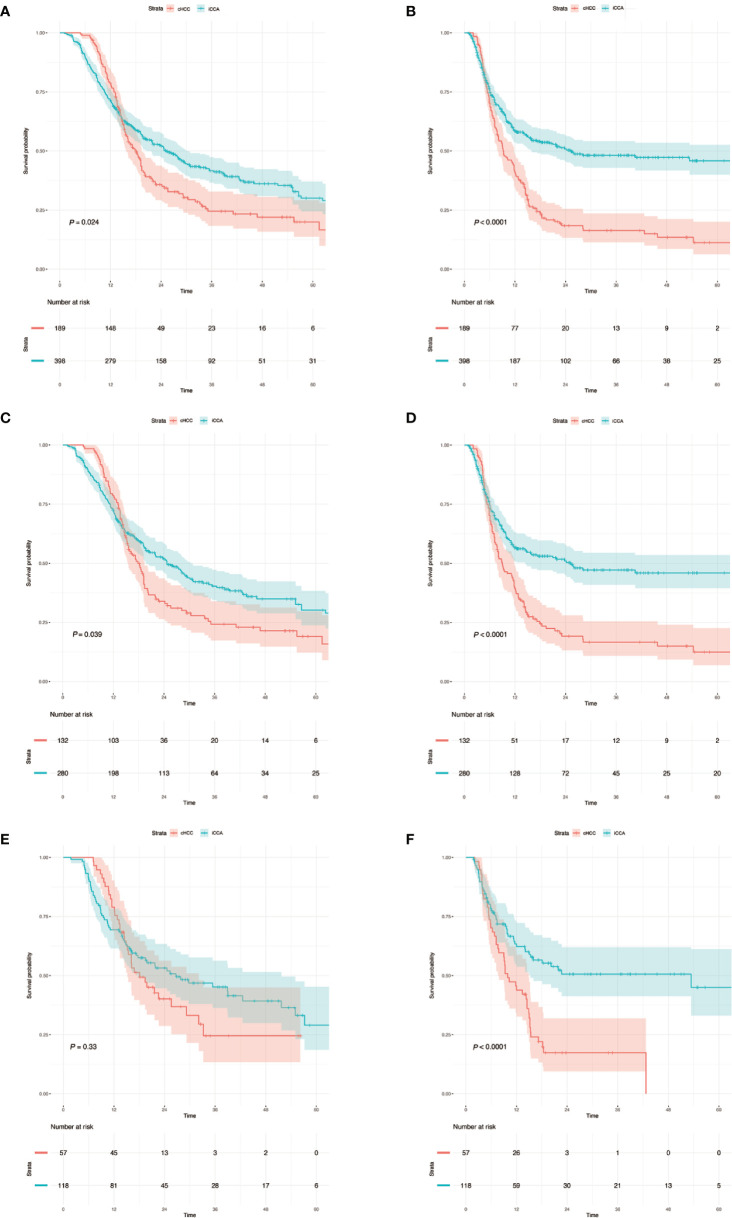
**(A, C, E)** Kaplan–Meier analysis of overall survival for cHCC and iCCA in the whole sets, training sets and validation sets. **(B, D, F)** Kaplan–Meier analysis of recurrence-free survival and overall survival for cHCC and iCCA in the whole sets, training sets and validation sets. The number at risk refers to the number of patients who have not relapsed at the corresponding time point. cHCC, combined hepatocellular cholangiocarcinoma; iCCA, intrahepatic cholangiocarcinoma.

A total of 587 patients (361 men, 226 women) who underwent curative liver resection for iCCA and cHCC between January 2008 and December 2017 at West China Hospital were enrolled in this study. All iCCA and cHCC patients were followed up after initial treatment until December 2019. In the training set, a total of 412 patients, including 132 cHCC patients and 280 iCCA patients. For the validation set, 175 consecutive patients were studied, consisting of 57 cHCC patients and 118 iCCA patients. There were more males in the cHCC group than in the iCCA group. Portal hypertension and HBsAg positivity were more common in patients with cHCC. However, microvascular invasion (MVI) and lymph node metastasis were more common in the iCCA group. Patients with cHCC were younger and had higher serum alpha-fetoprotein (AFP) levels and lower serum CA19-9 levels than patients with iCCA. The baseline clinicopathologic characteristics of the patients are listed in **Supplementary Table 1**. Among the entire set, the median overall survival (OS) of 189 patients with cHCC was 16.2 months and that of the 398 patients with iCCA was 18.6 months. The patients in the cHCC group had poorer OS and RFS than those in the iCCA group. The 1-, 3-, and 5-year OS rates were 78.3%, 12.2%, and 3.2%, respectively, in patients with cHCC and 70.1%, 23.1%, and 7.8%, respectively, in patients with iCCA (**Figure 2**).

### Constructing a Simplified Prediction Model and Internal Validation

**Figure 3:**
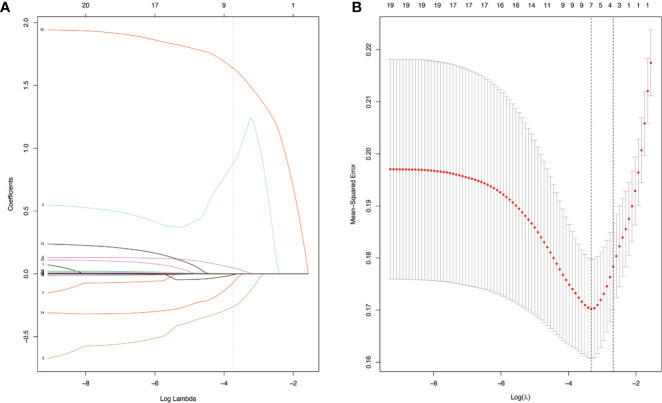
Blood-cHCC score models selection using the least absolute shrinkage and selection operator (LASSO) binary logistic regression model in the training set **(A)**. LASSO coefficient profiles of the seven selected blood signatures for combined hepatocellular cholangiocarcinoma (cHCC). A dashed vertical line is drawn at the value (logγ=-3.3) chosen by 10-fold cross-validation. Vertical line was shown at the value selected using cross-validation, where the optimum lambda gave rise to seven features with nonzero coefficients **(B)**. Partial likelihood deviance for the LASSO coefficient profiles. The partial likelihood deviance (binomial deviance) curve was presented versus log (lambda). A light dashed vertical line stands for the minimum partial likelihood deviance. A dashed vertical line stands for the partial likelihood deviance at the value (logγ=-3.3).

**Figure 4:**
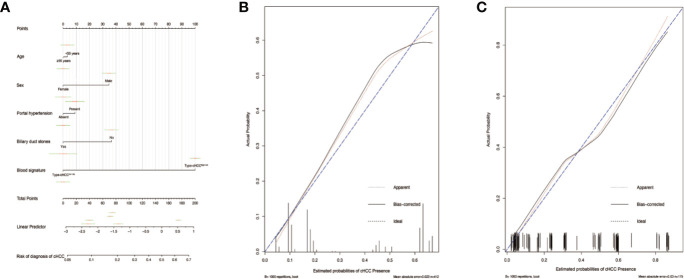
Differential diagnosis nomograms for distinguishing combined hepatocellular cholangiocarcinoma (cHCC) and intrahepatic cholangiocarcinoma (iCCA) and the calibration plot **(A)**. The nomogram maps the distinguishing ability of cHCC and iCCA on a scale of 0 to 200. For each covariate, a vertical line is drawn upwards and the corresponding points are noted. This is repeated for each covariate, ending with a total point that corresponds to the differential diagnosis axes to seek the probability of cHCC at the bottom of the nomogram. The C-index value for the nomogram distinguishing cHCC from iCCA was 0.796 (95% CI, 0.752–0.840). The calibration curve for distinguishing cHCC and iCCA in the training set **(B)** and in the validation set **(C)**. Ideal line (blue), estimated probabilities correspond to the actual observed; apparent line (red), prediction capability of the model obtained after data analysis; bias-corrected line, prediction capability of the model obtained after bootstrap correction.

**Figure 5:**
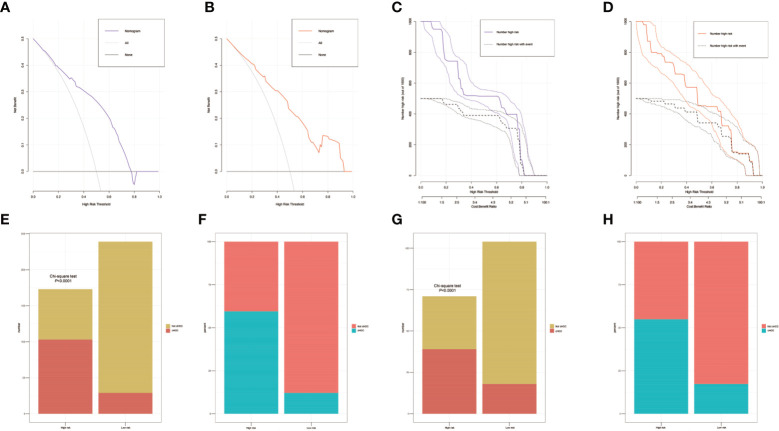
The decision curves of the nomograms for distinguishing combined hepatocellular cholangiocarcinoma (cHCC) from intrahepatic cholangiocarcinoma (iCCA) in the training and validation sets. The Y-axis represents the net benefit. The X-axis shows the threshold probability. The horizontal solid black line represents the hypothesis that no patients experienced presence of cHCC, and the solid gray line represents the hypothesis that all patients met the endpoint **(A, B)**. Clinical impact curves of the nomogram for distinguishing cHCC from iCCA in the training and validation set **(C, D)**. At different threshold probabilities within a given population, the number of high-risk patients and the number of high- risk patients with the outcome are shown. Discriminatory power of the nomograms for cHCC status with bar charts. Risk classification of the differential diagnosis nomogram conducted by the Receiver operating characteristic (ROC) curve analysis, and the performance in distinguishing the cHCC from iCCA in the training and validation set **(E–H)**.

To distinguish between cHCC and iCCA well before surgery and guide clinical decision making, univariate logistic regression analysis was performed to estimate the impacts of demographics, comorbid illnesses, and imaging features on the differential diagnosis of cHCC and iCCA in the training set. According to univariable logistic regression analysis, age, sex, biliary duct stones, and portal hypertension were associated with the possibility of cHCC diagnosis. Stepwise multivariate logistic regression analysis was further performed to identify significant independent risk factors. The multivariate analyses revealed that age (≥55 vs. <55 years, OR, 0.568, 95%CI, 0.366–0.882, *P* =0.012), sex (female vs. male, OR, 0.354, 95%CI, 0.215–0.582, *P* <0.001), biliary duct stones (yes vs. no, OR, 0.274, 95% CI, 0.103-0.729, *P*=0.010), and portal hypertension (present vs. absent, OR, 1.816, 95% CI, 1.066–3.095, *P*=0.028) were independent risk factors for distinguishing cHCC from iCCA (**Table 2**). Using the coefficients derived from the LASSO logistic regression models in the training set, we then constructed a formula to calculate for each patient. The LASSO coefficient profiles of the selected blood features are shown in **Figure 3**. The blood signature score was based on the personalized levels of the 7 blood features, as listed in **Supplementary Table 2**. Restrictive cubic spline functions of the blood-cHCC scores in the training and validation sets showed that the blood-cHCC score presented linear profiles (**Supplementary Figure 2**). Using the ROC curve, we classified patients into a type-cHCC^low risk^ group and a type-cHCC^high risk^ group with a blood signature score of -0.535 as the cut-off value (**Supplementary Figure 1A**). Based on the results of the blood signatures and multivariate logistic regression, a nomogram for distinguishing cHCC and iCCA was established (**Figure 4**). Point assignments and differential scores for each variable in the nomogram models are presented in **Supplementary Table 3**. According to the nomogram for distinguishing between cHCC and iCCA, the blood signature made the largest contribution. The calibration curve of the prediction nomogram for the differential diagnosis of cHCC and iCCA presented a good agreement in training and validation sets (**Figures 4B, C**). The Harrell’s concordance index (C-index) for the nomogram for distinguishing cHCC and iCCA was 0.796 (95% CI, 0.752–0.840) for the training set and 0.824 (95% CI, 0.761–0.887) for the validation set, as detailed in **Supplementary Table 4**. After obtaining risk scores were obtained from the nomogram, risk classification of the differential diagnosis nomogram was conducted by ROC curve analysis. The patients were classified into low- and high-risk groups according to the optimal cut-off score of 119 on the cHCC nomogram (**Supplementary Figure 1B**). The high-risk group had a noticeably increased possibility of cHCC in the training set and validation set (**Figures 5E–H**). In addition, we performed survival analysis based on the cHCC nomogram risk score, and the high-risk groups had a worse prognosis in terms of RFS (**Supplementary Figure 3**). Hence, the nomogram could effectively distinguish between cHCC and iCCA before surgery but also predict prognosis after surgery to some extent.

**Table 2:** Logistic regression models of variables associated with distinguish combined hepatocellular cholangiocarcinoma (cHCC) from intrahepatic cholangiocarcinoma (iCCA) before surgery.

Variable	Univariate regression model			Multivariate regression model		
	Odds ratio	95% CI	*P* value	Odds ratio	95% CI	*P* value
Age (≥55 vs. <55 years)	**0.530**	**0.348**–**0.805**	**0.003**	**0.568**	**0.366**–**0.882**	**0.012**
Sex (female vs. male)	**0.301**	**0.186**–**0.488**	<**0.001**	**0.354**	**0.215**–**0.582**	**<0.001**
Diabetes (yes vs. no)	0.887	0.482–1.631	0.700			
Portal hypertension (present vs. absent)	**2.020**	**1.204**–**3.389**	**0.008**	**1.816**	**1.066**–**3.095**	**0.028**
Biliary duct stones (yes vs. no)	**0.206**	**0.080**–**0.531**	**0.001**	**0.274**	**0.103**–**0.729**	**0.010**
Maximum tumor size (>5 vs. ≤5cm)	0.900	0.593–1.365	0.620			
Tumor number (multiple vs. single)	0.792	0.501–1.251	0.317			
Macroscopic vascular invasion (yes vs. no)	0.746	0.475–1.174	0.206			

cHCC, combined hepatocellular carcinoma; iCCA, intrahepatic cholangiocarcinoma; CI, confidence interval.

Bold indicates statistically significant difference.

## Discussion

Previous studies have classified cHCC and iCCA in the same category (7, 21), however, the prognosis of cHCC in comparison to iCCA remains controversial. Furthermore, treatment strategies for cHCC and iCCA differ. A previous study demonstrated that the molecular biology features of cHCC are more similar to those of hepatocellular carcinoma (HCC) than of iCCA, therefore, multitargeted inhibitors, including lenvatinib, regorafenib, and cabozantinib, may have potential for benefit in cHCC due to frequent alterations in RTK/Ras/PI3‐kinase pathways (22). Moreover, liver transplantation might also bring potential survival benefits to patients with cHCC (23, 24). However, due to the current controversy over the value of liver transplantation for iCCA and the lack of indications for liver transplantation that meet the characteristics of the disease, liver transplantation has not been recommended as a routine treatment for iCCA. Since iCCA often metastasizes to distant sites in the early stage of the disease, which seriously affects long-term survival after transplantation, most centers in the West hold a conservative attitude towards the treatment of liver transplantation for iCCA (25). Curative liver resection is an important treatment for two types of resectable tumors. For resectable tumor, if we can distinguish the pathological type of the tumor before surgery, we can perform a comprehensive assessment to choose a wider surgical margin, anatomic liver resection. we could choose individualized treatment for each patient’s condition especially for unresectable patients or patients who could not guarantee the R0 margin of surgery, which is in line with the current concept of precision liver cancer surgery (26). cHCC is associated with high risk of recurrence following surgical resection as compared with iCCA. Closely post-operative monitoring is highly recommended for cHCC patients. Simultaneously, it could aid clinicians in explaining the illness for patient counseling. For unresectable iCCA, chemotherapy with gemcitabine, platinum compounds, and fluoropyrimidines is the main treatment choice. A recent multicenter study reported that postoperative chemotherapy with gemcitabine prolonged the survival time of patients at high risk of recurrence and metastasis (27). However, systemic therapy or chemotherapy is not the standard option for advanced and unresectable cHCC (28), and a large sample size is still needed to distinguish cHCC from iCCA, and determine the value of other treatments for cHCC, which reflected the significance and importance of our research.

To data, the gold standard for the preoperative diagnosis of liver tumors is fine needle aspiration biopsy, but for tumors without a biopsy path or with a small-diameter tumors, biopsies are usually not available before surgery. In addition, the pathological data obtained at the morphological, phenotypical, and molecular levels from these tiny fragments by fine-needle aspiration biopsy may be incomplete or only partially representative, especially for cHCC patients with two components. The real risk of seeding and the oncologic prognosis by inserting a needle into a liver tumor lesion are still unclear (29–31). In recent years, with the development of imaging technology, the role of liver biopsy in the diagnosis of primary liver cancer (PLC) has been challenged over time by the ability of imaging techniques to conjecture the histologic status (32, 33). Imaging techniques could also help clinicians to understand more information, such as vascular invasion and lymph node metastasis, and even determine the most appropriate operative method (34). However, the diagnosis of cHCC and the differentiation of cHCC from other PLCs based on imaging findings can be challenging because of the histologic diversity and complexity of cHCC components and the overlapping imaging characteristics with those of iCCA (35–37). Moreover, their clinical value is limited due to the lack of costly high-resolution equipment and experienced radiologists especially in some developing areas. Therefore, a novel and noninvasive method is required to distinguish cHCC from iCCA before receiving various treatments.

To our knowledge, our research is the first large comprehensive comparison reported to date on the clinical characteristics and prognoses of cHCC and iCCA patients after surgery. Our study focused on distinguishing between cHCC and iCCA before liver resection using a simple predictive model that incorporated the clinical risk factors as well as laboratory blood indicators that could be used in daily clinical practice to accurately predict pathological information preoperatively, rather than being limited to the identification of clinicopathological risk factors in resected specimens.

In this study, we showed that the prognosis of cHCC was significantly worse than that of iCCA in both the training set and in the validation set. Differences in prognosis for iCCA and cHCC might be due to their distinct mechanisms of carcinogenesis and biological behaviors. It is increasingly believed that cHCC may originate from hepatic progenitor cells, which are intermediate stem cells capable of undergoing bidirectional differentiation into hepatocytes and bile duct epithelial cells (38, 39), causing cHCC to have significant heterogeneity and aggressive biological behavior. Coulouarn et al. determined that the occurrence of cHCC might be related to the microenvironment remodeling and the activation of TGFβ and Wnt/β-catenin were identified as the two major signaling pathways in cHCC (39). In addition, most patients with cHCC have a background of hepatitis B cirrhosis, which easily leads to tumor recurrence. However, for iCCA, the possible causative risk factors include biliary diseases such as biliary duct stones, hepatobiliary flukes, primary sclerosing cholangitis, and biliary tract cysts (40). Although our study found that the cHCC and iCCA groups had significant differences in MVI and lymph node metastasis, which might be the basis for distinguishing between cHCC and iCCA, the above information was obtained only through postoperative pathological specimens. In our study, we found that liver function and coagulation function indicators in cHCC patients were higher than those in iCCA patients which might be related to the facts that cHCC patients usually are infected with hepatitis virus. The above might become a potential blood predictor to distinguish cHCC from iCCA. Age (<55 years) and portal hypertension were positively related to cHCC, while biliary duct stones and female sex were positive factors in the iCCA differential nomogram. Our differential diagnosis nomogram demonstrated good agreement between predictions and observations in the training and validation sets. In addition, we found that the nomogram we established has better diagnostic performance than other clinical risk factors or blood signatures alone. With our nomogram, we can identify the cHCC patients who were previously misdiagnosed with iCCA. These patients could regain the chance to undergo liver transplantation or targeted therapy. Additionally, our nomogram might serve as a selection tool to assess neoadjuvant treatment for iCCA patients during randomized clinical trials in the future. Meaningfully, the Kaplan–Meier survival curves demonstrated that the nomograms could not only effectively distinguish between cHCC and iCCA regardless of individual values, but also successfully discriminate among different risk groups, thereby improving clinical decision making.

Although our research provided a new and simple method to distinguish between iCCA and cHCC, several limitations should be taken into consideration when interpreting our findings. Our study was conducted at a single-center study, and due to the characteristics of retrospective studies, there may be potential selection bias. The data for the training set and validation set were obtained from a single center, which might have hampered the identification of possibly important predictive factors. Moreover, although hepatitis virus infection is an important pathogenic factor in the carcinogenesis of PLC in China, however, in the West, hepatitis C virus infection and alcohol or metabolic factors are usually the causes of PLC. Whether this differential diagnosis nomogram is generalizable to patients in Western countries is still worth exploring, other western liver cancer centers are needed to recruit to build external validation.

## Conclusion

In conclusion, the current study proves that patients with cHCC have a poorer prognosis than those with iCCA and that cHCC is a distinct tumor different from iCCA. Furthermore, we constructed and validated a nomogram that optimally differentiates cHCC from iCCA preoperatively by combining other clinical risk factors identified by logistic regression and blood signatures selected by the LASSO algorithm.

## Data Availability Statement

The raw data supporting the conclusions of this article will be made available by the authors, without undue reservation.

## Ethics Statement

The study was approved by ethics committee of Sichuan University West China hospital and informed consent was taken from all the patients.

## Author Contributions

Study concept and design: TW, WXW, and WTW. Acquisition of data: TW, SS, and JZ. Statistical analysis of data: TW and XY. Drafting of the manuscript: TW and WXW. Critical revision of the manuscript: TW, WXW, SS, and WTW. All authors contributed to the article and approved the submitted version.

## Funding

This research was funded by the National Natural Science Foundation of China (No. 81770566) and the Department of Science and Technology of Sichuan Province (No. 19ZDYF1682).

## Conflict of Interest

The authors declare that the research was conducted in the absence of any commercial or financial relationships that could be construed as a potential conflict of interest.

## Supplementary Material

Supplementary Figure 1
**(A)** Receiver operating characteristic (ROC) curve analysis of the blood signature for cHCC. The areas under the curve (AUCs) were 0.806. The optimal cut-off blood score was -0.535 **(B)** Receiver operating characteristic (ROC) curve analysis of the nomogram risk score for distinguishing cHCC. The areas under the curve (AUCs) were 0.799. The optimal cut-off the nomogram risk score was 119.

Supplementary Figure 2 The restricted cubic spline of the blood-cHCC score in training and validation sets **(A, B)**. The restricted cubic spline of the nomogram risk score for distinguishing cHCC in training and validation sets **(C, D).**

Supplementary Figure 3 Kaplan–Meier analysis of recurrence-free survival and overall survival according to risk score based on nomogram. The number at risk refers to the number of patients who have not relapsed at the corresponding time point.
