# Supplementary figures and images for: Development and Validation of a Nomogram for Differentiating Combined Hepatocellular Cholangiocarcinoma From Intrahepatic Cholangiocarcinoma
Source: Front Oncol. 2020 Dec 9;10:598433. doi: 10.3389/fonc.2020.598433 (PMC7756117; doi:10.3389/fonc.2020.598433)

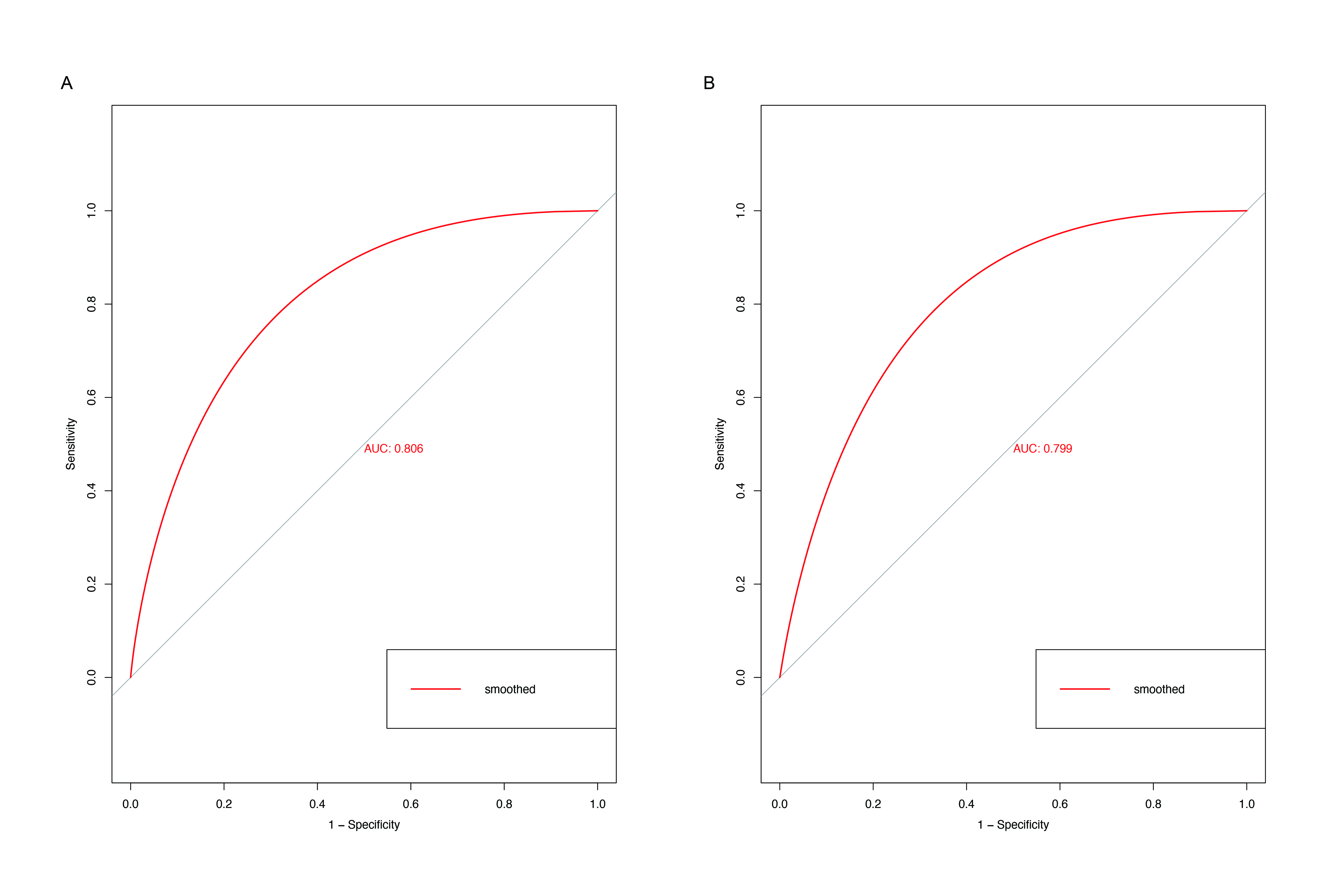

Supplement: Supplementary Figure 1 — (A) Receiver operating characteristic (ROC) curve analysis of the blood signature for cHCC. The areas under the curve (AUCs) were 0.806. The optimal cut-off blood score was -0.535 (B) Receiver operating characteristic (ROC) curve analysis of the nomogram risk score for distinguishing cHCC. The areas under the curve (AUCs) were 0.799. The optimal cut-off the nomogram risk score was 119. [file Image_1.tif]

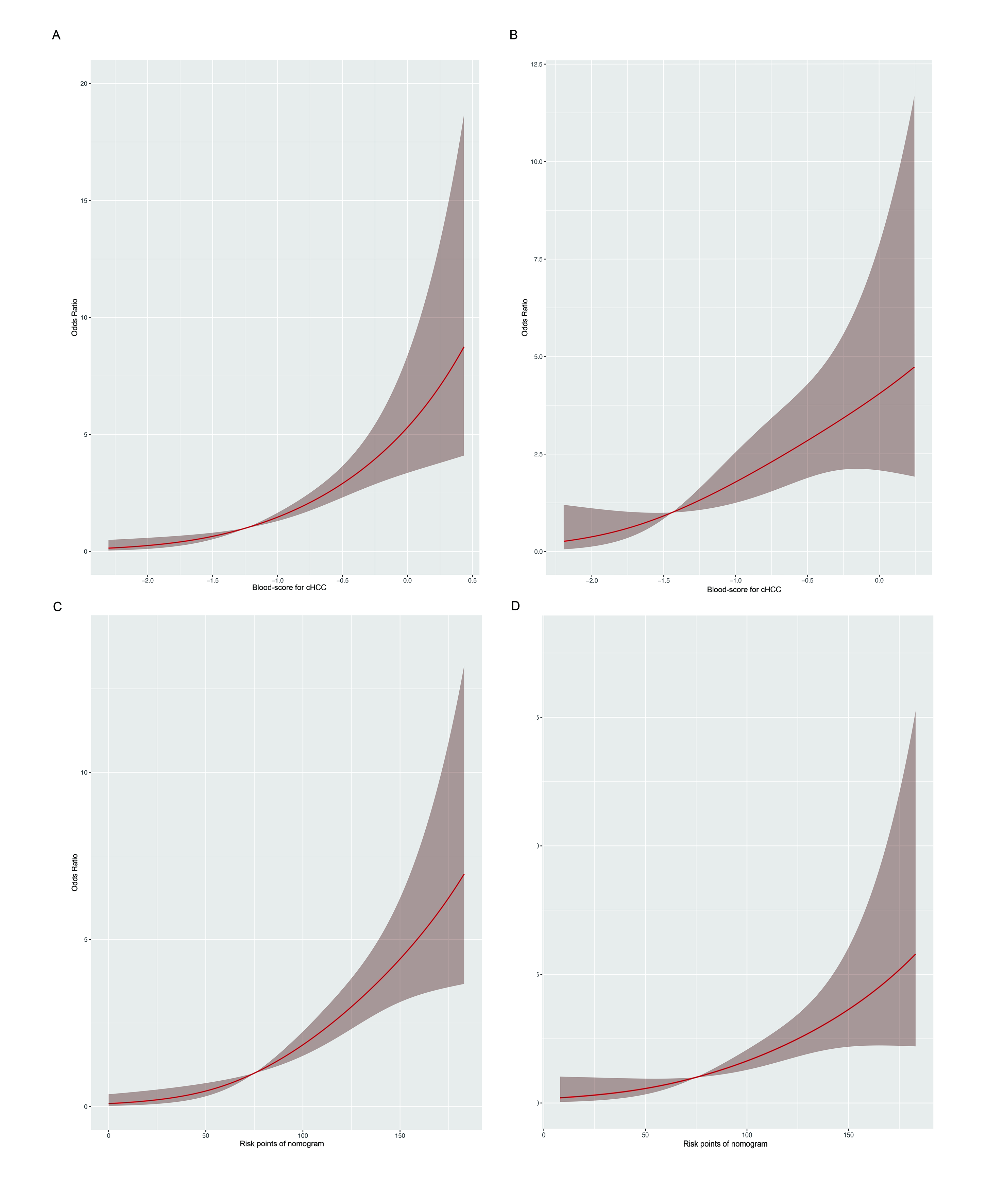

Supplement: Supplementary Figure 2 — The restricted cubic spline of the blood-cHCC score in training and validation sets (A, B). The restricted cubic spline of the nomogram risk score for distinguishing cHCC in training and validation sets (C, D). [file Image_2.tif]

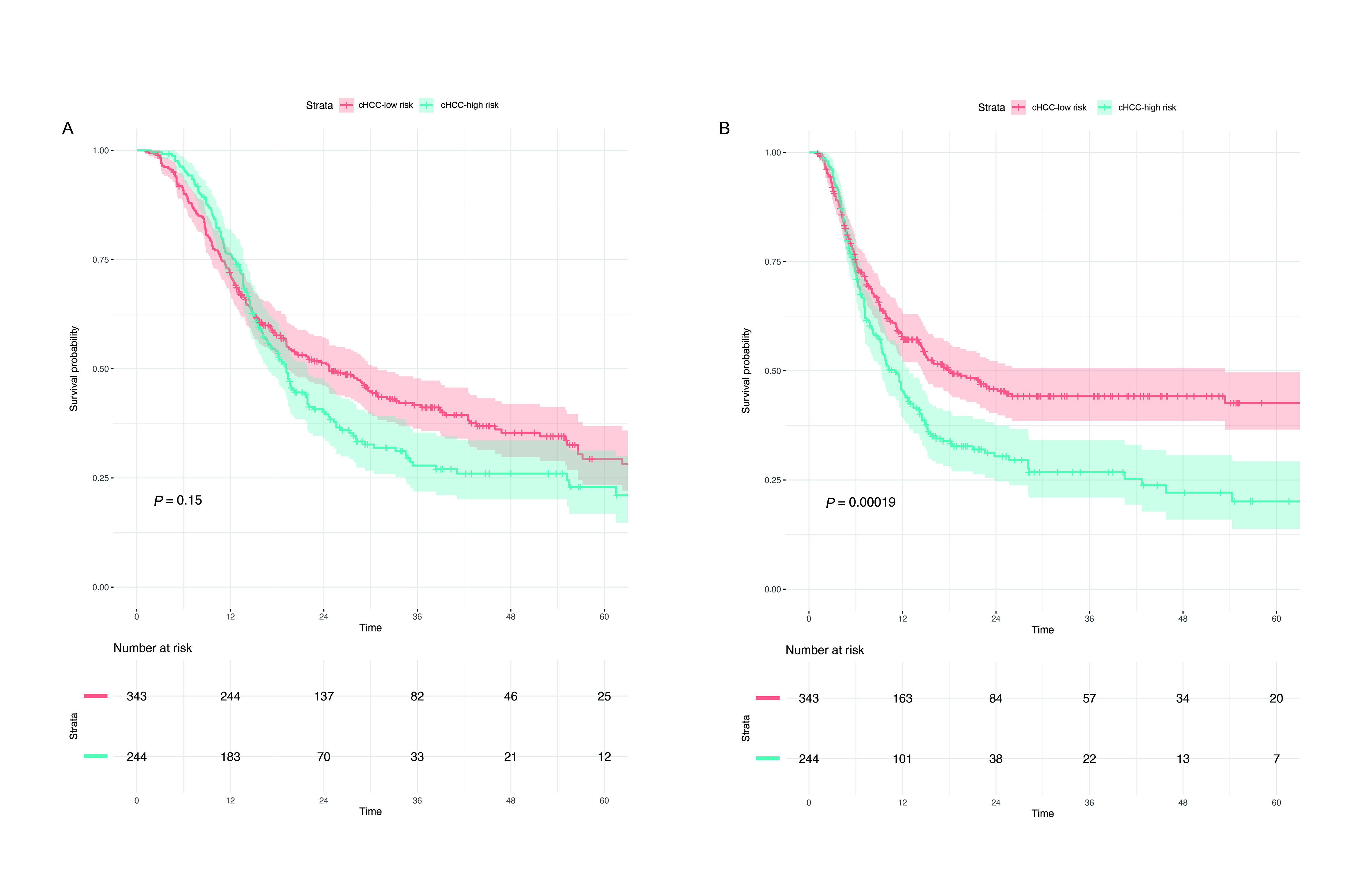

Supplement: Supplementary Figure 3 — Kaplan–Meier analysis of recurrence-free survival and overall survival according to risk score based on nomogram. The number at risk refers to the number of patients who have not relapsed at the corresponding time point. [file Image_3.tif]
