# Supplementary material for: Development and Validation of a Nomogram for Differentiating Combined Hepatocellular Cholangiocarcinoma From Intrahepatic Cholangiocarcinoma
Source: Front Oncol. 2020 Dec 9;10:598433. doi: 10.3389/fonc.2020.598433 (PMC7756117; doi:10.3389/fonc.2020.598433)
Supplement: Supplementary file 4 [file Table_1.docx]

**Supplementary Table 1. The comparison of clinicopathological factors between cHCC and iCCA group**

| **Characteristic** | **Patients** | | ***P* value** |
| --- | --- | --- | --- |
|  | **iCCA(n=398)** | **cHCC(n=189)** |  |
| **Age (years), median (IQR)** | 57 (49-64) | 52(42-59) | <0.001 |
| **Gender, (male/ female)** | 206/192 | 155/34 | <0.001 |
| **Portal hypertension, n (%)** |  |  | <0.001 |
| Yes | 53(13.3%) | 53(28.0%%) |  |
| No | 345(86.7%) | 136(72.0%) |  |
| **HBsAg positive, n (%)** |  |  | <0.001 |
| Yes | 126(31.7%) | 145(76.7%) |  |
| No | 272(68.3%) | 44(23.3%) |  |
| **Diabetes, n (%)** |  |  | 0.438 |
| Yes | 51(12.8%) | 20(10.6%) |  |
| No | 347(81.2%) | 169(89.4%) |  |
| **Biliary duct stones, n (%)** |  |  | <0.001 |
| Yes | 66(16.6%) | 8(4.2%) |  |
| No | 332(83.4%) | 181(95.8%) |  |
| **Baseline laboratory investigations** |  |  |  |
| WBC count ×10^9^/L, median (IQR) | 6.62 (5.41-7.92) | 5.77 (4.73-7.24) | 0.001 |
| NEUT count ×10^9^/L, median (IQR) | 4.24 (3.30-5.58) | 3.59(2.87-5.11) | 0.003 |
| PLT count ×10^9^/L, median (IQR) | 167(123.75-218.5) | 140(92-184) | <0.001 |
| ALT (U/L), median (IQR) | 27 (18-43) | 34(26-52) | <0.001 |
| AST (U/L), median (IQR) | 30(23-40) | 35(27-52) | <0.001 |
| GGT (U/L), median (IQR) | 66(34-133) | 66(39.5-132.5) | 0.977 |
| TBIL (umol/L), median (IQR) | 11.7 (9.68-16.8) | 13.4 (10-18.65) | 0.232 |
| ALB (g/L), median (IQR) | 42.75(39.98-45.3) | 42.1(38.45-45.15) | 0.148 |
| PT(s), median (IQR) | 11.6 (11.0-12.3) | 12.0(11.35-12.9) | 0.002 |
| INR, median (IQR) | 1.01(0.96-1.08) | 1.04(1.00-1.135) | <0.001 |
| AFP, ng/ml median (IQR) | 3.20(2.22-5.44) | 57.33(4.62-481.25) | <0.001 |
| CA19-9 level(U/mL), median (IQR) | 78.29(17.82-748.2) | 29.27(14.33-85.74) | <0.001 |
| **Tumor size (cm), median (range)** | 5.85(4.2-8) | 5.5(3.65-7.55) | 0.299 |
| **Tumor number, (Multiple/solitary)** |  |  | 0.011 |
| multiple | 129(32.4%) | 42(22.2%) |  |
| solitary | 269(67.6%) | 147(77.8%) |  |
| **Tumor location, n(%)** |  |  | <0.001 |
| Left lobe | 162(40.7%) | 49 (25.9%) |  |
| Right lobe | 147(36.9%) | 117 (61.9%) |  |
| Both lobes | 89(22.4%) | 23(12.2%) |  |
| **Extent of liver resection, n(%)** |  |  | 0.338 |
| major | 247(62.1%) | 125(66.1%) |  |
| minor | 152(38.2%) | 64(33.9%) |  |
| **MVI, n (%)** |  |  | <0.001 |
| Yes | 73 (18.3%) | 66 (34.9%) |  |
| No | 325 (81.7%) | 123 (65.1%) |  |
| **Macroscopic vascular invasion, n (%)** |  |  | 0.425 |
| Yes | 131 (32.9%) | 56(29.6%) |  |
| No | 267 (67.1%) | 133(70.4%) |  |
| **Satellite nodules, n (%)** |  |  | 0.259 |
| Yes | 63(15.8%) | 37(19.6%) |  |
| No | 335(84.2%) | 152(80.4%) |  |
| **Lymph node metastasis, n (%)** |  |  | 0.002 |
| Present | 102 (25.6%) | 27(14.3%) |  |
| Absent | 296 (74.4%) | 162(85.7%) |  |
| **Tumor encapsulation, n (%)** |  |  | 0.238 |
| incomplete | 227(57%) | 98(51.9%) |  |
| complete | 171(43%) | 91(48.1%) |  |

**Abbreviations:** *cHCC* combined hepatocellular cholangiocarcinoma; *iCCA* intrahepatic cholangiocarcinoma; *HBsAg* hepatitis B surface antigen; *WBC* white blood cell; *NEU* neutrophil; *PLT* platelet; *ALT* alanine aminotransferase; *AST* aspartate transaminase; *GGT* γ-glutamyl transferase; *TBIL* total bilirubin; *ALB* albumin; *PT* Prothrombin time; *INR* international normalized ratio; *AFP,* alpha fetoprotein; *CA19-9* carbohydrate antigen 19-9; *MVI* microvascular invasion;
