# Supplementary material for: Development and Validation of a Nomogram for Differentiating Combined Hepatocellular Cholangiocarcinoma From Intrahepatic Cholangiocarcinoma
Source: Front Oncol. 2020 Dec 9;10:598433. doi: 10.3389/fonc.2020.598433 (PMC7756117; doi:10.3389/fonc.2020.598433)
Supplement: Supplementary file 5 [file Table_2.docx]

**Supplementary Table 2. Coefficients for each blood index in the LASSO regression models for cHCC status.**

| **cHCC presence** | |
| --- | --- |
| **Index** | **coefficient** |
| LYM | -1.6507 E-01 |
| INR | 1.16152 |
| PT | 1.3087E-02 |
| AST | 4.2790E-04 |
| AFP | 4.0029E-05 |
| CA19-9 | -6.8105E-04 |
| HbsAg | 1.52329 |

**Abbreviations:** *cHCC* combined hepatocellular cholangiocarcinoma; *LASSO* Least Absolute Shrinkage and Selection Operator; *LYM* lymphocyte; *INR* international normalized ratio; *PT* Prothrombin Time; *AST* aspartate transaminase; *AFP,* alpha fetoprotein; *CA19-9* carbohydrate antigen 19-9; *HbsAg* hepatitis B surface antigen；
