# Supplementary material for: Development and Validation of a Nomogram for Differentiating Combined Hepatocellular Cholangiocarcinoma From Intrahepatic Cholangiocarcinoma
Source: Front Oncol. 2020 Dec 9;10:598433. doi: 10.3389/fonc.2020.598433 (PMC7756117; doi:10.3389/fonc.2020.598433)
Supplement: Supplementary file 6 [file Table_3.docx]

**Supplementary Table 3 Point assignments and differential diagnosis scores for each variable in the nomogram models.**

| Variables | Classification | Nomogram risk score^*^ |
| --- | --- | --- |
| Age | <55 years | 3 |
|  | ≥55 years | 0 |
| Sex | Male | 35 |
|  | Female | 0 |
| Portal hypertension | Present | 8 |
|  | Absent | 0 |
| Biliary duct stones | Yes | 0 |
|  | No | 37 |
| Blood signature | Type-cHCC^high risk^ | 100 |
|  | Type-cHCC^low risk^ | 0 |

^*^Nomogram risk score for distinguishing cHCC from iCCA
