# Supplementary material for: Development and Validation of a Nomogram for Differentiating Combined Hepatocellular Cholangiocarcinoma From Intrahepatic Cholangiocarcinoma
Source: Front Oncol. 2020 Dec 9;10:598433. doi: 10.3389/fonc.2020.598433 (PMC7756117; doi:10.3389/fonc.2020.598433)
Supplement: Supplementary file 7 [file Table_4.docx]

**Supplementary Table 4 Predictive accuracy of the nomograms for distinguishing between cHCC and iCCA**

| **Model** | **Group** | **Variable** | **AUC/C-index** | **Sensitivity (%)** | **Specificity (%)** | **Positive predictive value (%)** | **Negative predictive value (%)** |
| --- | --- | --- | --- | --- | --- | --- | --- |
| Distinguish cHCC from iCCA | Training | Nomogram | 0.796 (95% CI, 0.752- 0.840) | 78.03% | 75.00% | 59.54% | 87.87% |
|  |  | others^*^ | 0.696(95% CI, 0.645- 0.747) | 59.85% | 72.50% | 50.64% | 79.30% |
|  |  | Blood signature | 0.762(95% CI,0.718-0.805) | 78.03% | 74.29% | 58.86% | 87.76% |
|  | Validation | Nomogram | 0.824 (95% CI, 0.761- 0.887) | 82.46% | 67.80% | 55.29% | 88.89% |
|  |  | others^*^ | 0.799(95% CI, 0.732- 0.866) | 87.72% | 58.47% | 50.51% | 90.79% |
|  |  | Blood signature | 0.698 (95% CI, 0.625- 0.771) | 68.42% | 71.19% | 53.42% | 82.35% |

**Abbreviations:** *cHCC* combined hepatocellular cholangiocarcinoma; *iCCA* intrahepatic cholangiocarcinoma;

^*Others: clinical risk factors including demographic, comorbid illnesses and imaging features.^
